# The Key Role of Nutritional Intervention in Delaying Disease Progression and the Therapeutic Management of Diabetic Kidney Disease—A Challenge for Physicians and Patients

**DOI:** 10.3390/jpm14080778

**Published:** 2024-07-23

**Authors:** Ileana Peride, Miruna Anastasiu, Silvia Alexandra Serban, Mirela Tiglis, Razvan Ene, Ana-Maria Nechita, Tiberiu Paul Neagu, Ionel Alexandru Checherita, Andrei Niculae

**Affiliations:** 1 Clinical Department No. 3, “Carol Davila” University of Medicine and Pharmacy, 020021 Bucharest, Romania; niculaeandrei@yahoo.com; 2 “Marie Skłodowska Curie” Children Emergency Clinical Hospital, 077120 Bucharest, Romania; 3 Emergency Clinical Hospital of Bucharest, 014461 Bucharest, Romania; 4 Department of Anesthesia and Intensive Care, Emergency Clinical Hospital of Bucharest, 014461 Bucharest, Romania; 5 Clinical Department No. 14, “Carol Davila” University of Medicine and Pharmacy, 020021 Bucharest, Romania; 6 Department of Nephrology and Dialysis, “St. John” Emergency Clinical Hospital, 042122 Bucharest, Romania; 7 Clinical Department No. 11, “Carol Davila” University of Medicine and Pharmacy, 050474 Bucharest, Romania; dr.neagupaul@gmail.com; 8 Department of Nephrology, Ilfov County Emergency Clinical Hospital, 022104 Bucharest, Romania; al.checherita@gmail.com

**Keywords:** chronic kidney disease, diabetic kidney disease, nutrition regimen, outcome

## Abstract

Chronic kidney disease (CKD) represents an increasingly common pathology that affects patients’ quality of life, and it is frequently associated with a high mortality rate, especially in the final stages of the disease. At the same time, diabetes mellitus is a chronic disease that contributes to the increased number of patients with CKD through diabetic kidney disease (DKD). The alternation of hypoglycemia with hyperglycemia is a condition in the occurrence of microvascular complications of diabetes, including DKD, which involves structural and functional changes in the kidneys. The therapeutic management of diabetic nephropathy is a much-discussed topic, both from nutritional medical recommendations and a pharmacotherapy perspective. The diet starting point for patients with DKD is represented by a personalized and correct adjustment of macro- and micronutrients. The importance of nutritional status in DKD patients is given by the fact that it represents a modifiable factor, which contributes to the evolution and prognosis of the disease. Since, in most cases, it is necessary to restrict many types of food, malnutrition must be considered and avoided as much as possible.

## 1. Introduction

Chronic kidney disease (CKD) is a global health problem, and the number of patients with this disease is continuously increasing. A meta-analysis of observational studies estimating CKD prevalence showed that approximately 13.4% of the world’s population has CKD [1]. Diabetic nephropathy, a complication characterized by persistent microalbuminuria and a progressive decline in renal function, is strongly influenced by poor control of glycemic values and the duration of diabetes.

In 2007, the National Kidney Foundation (NKF) introduced new terminology, diabetic kidney disease (DKD), because the diagnosis of renal impairment due to diabetes mellitus (DM) is based on clinical and laboratory assessments. The term diabetic nephropathy implies that a renal biopsy has been performed, which is rarely carried out in daily practice [2].

The estimated number of diabetic patients with CKD has reached 24.3 million in China [3]. In the United States, diabetic patients represent 30% to 50% of end-stage kidney disease cases [4]. In 2015, the International Diabetic Federation reported that almost 440 million patients between the ages of 20 and 79 were diabetic, representing a prevalence of approximately 8.8%; by 2035, this number could increase to 550 million individuals, and by 2040, the number of patients with type 1 DM is expected to reach more than 17 million cases [5,6,7]. From the moment of their first medical visit, renal failure (CKD stages 3–5) was noted in 21.6% of type 2 diabetic patients, according to the ADD-CKD study [8].

An important factor in the evolution of patients with DKD is represented by a properly adjusted diet according to the entire existing pathology [9]. Usually, screening of renal function, including albuminuria, should be performed at the moment of diagnosis and annually thereafter in type 2 diabetes patients and after 5 years from diagnosis in type 1 DM patients. According to new data that showed an albuminuria prevalence of almost 18% in type 1 DM patients before 5 years from diagnosis, renal function screening will probably be required 1 year after diagnosis. In type 2 DM patients, microalbuminuria was noted in 7% of patients at the moment of diagnosis [10].

Early renal damage is characterized by increased albumin excretion. Initially, microalbuminuria (30 to 299 mg/24 h) is noted, followed by macroalbuminuria (≥300 mg/24 h) in the advanced stages of the disease [11]. Microalbuminuria is an important test that contributes to the diagnosis of kidney disease; it can also suggest a chance of a reversible pathology, and some patients may regress to normoalbuminuria [12]. Typically, microvascular complications, including DKD, are diagnosed after approximately 15 years of diabetes [13]. In addition, the duration and severity of hyperglycemia are the major causes of renal damage. Therefore, the goal of DKD therapy consists of preventing increased urinary albumin excretion and kidney and cardiovascular event onset [14].

The current drug treatment recommended in DKD is primarily based on angiotensin-converting enzyme inhibitors (ACEIs) and angiotensin receptor blockers (ARBs) to block the renin–angiotensin–aldosterone system (RAAS) but also on sodium/glucose cotransporter 2 (SGLT2) inhibitors, glucagon-like peptide 1 (GLP-1) agonists, mineralocorticoid receptor antagonists, and endothelin antagonists, with effects both on glycemic control and RAAS blockade. However, nutritional status plays a significant role in the optimal control of glycemic values and proteinuria decrease [15].

## 2. Nutritional Medical Therapy in DKD

In any pathology, nutrition represents an important modifiable factor that can improve treatment management. The prevention of microalbuminuria or stopping progression to macroalbuminuria represents the primary goal of DKD treatment [16,17,18]. In contrast with the diabetic regimen, the DKD diet is slightly more complex and restrictive, as it should be adapted according to the patient’s stage of disease and associated comorbidities [19,20]. Therefore, the DKD diet should be designed not only to control blood glucose but also to delay the progression of kidney disease through the effective regulation of protein, sodium, and potassium levels. This will lead to adequate control or prevention of the onset of hypertension, uremia, edema, hyperkalemia, and hyperlipidemia [21].

It is advisable to advocate for nutritious dietary habits among CKD patients. This approach aims to prevent or alleviate these risk factors, ultimately reducing the likelihood of developing CKD. Nutritional interventions have shown the potential to lower glycated hemoglobin (HbA1c) levels comparably to or even more effectively than glucose-lowering medications. Simple dietary recommendations, such as increasing the consumption of non-starchy vegetables, reducing the intake of added sugars and refined grains, and opting for whole foods over heavily processed ones, can be implemented for the majority of patients. A diet rich in vegetables, fruits, whole grains, fiber, legumes, plant-based proteins, unsaturated fats, and nuts aligns with many dietary patterns associated with positive health outcomes in the general population. This approach serves as a suitable starting point for individuals with diabetes and CKD [22].

## 3. Caloric Intake

Several strategies can be utilized to mitigate insufficient nutrient consumption among individuals with chronic kidney disease. For clinically stable patients with CKD stages 3–5, who are not undergoing dialysis, it is essential to preserve their protein reserves. Therefore, it is recommended to maintain a dietary protein and energy intake within the range of 0.6–0.8 g of proteins per kilogram of ideal body weight per day and 30–35 kilocalories per kilogram of ideal body weight per day. However, adjustments to these levels are necessary during hypermetabolic conditions such as acute illness and hospitalizations. For elderly individuals with CKD, usually with a sedentary lifestyle, an energy intake of 30 kilocalories per kilogram of body weight per day is acceptable [23].

## 4. Protein Intake

From their first medical appointment, CKD patients should be educated on a healthy diet that is low in salt, low in saturated fats, high in fiber, and low in energy. Protein restriction should be progressively increased according to the stages of CKD. Additionally, physical activity should complement the nutritional treatment [24].

For individuals with an estimated glomerular filtration rate (eGFR) below 60 mL/min/1.73 m^2^ and who do not exhibit nephrotic syndrome, it is recommended to have a daily protein intake of 0.8 g/kg. Research on nutrition in patients with decreased eGFR suggests that protein consumption can be safely reduced to 0.6 g/kg/day [25,26,27,28,29], although adopting a very low-protein diet has been linked to increased long-term mortality risk, especially in older patients [29,30]. In addition, there are studies that concluded that low-protein diets did not improve renal impairment, eGFR, or 24 h urinary albumin excretion [25]. Moderate restriction is typically well-accepted, does not result in malnutrition among CKD patients, and is also helpful in avoiding metabolic acidosis. Evidence shows that a daily decrease in protein intake of 0.2 g per kilogram of body weight ameliorates metabolic acidosis, hyperphosphatemia, and the uremic state of these patients [24,31].

In CKD, the dietary protein requirement to maintain a neutral nitrogen balance is approximately 0.55–0.60 g/kg/day, with the condition of providing sufficient energy intake [32,33]. However, most patients on a low-protein diet consume more protein than recommended, rarely falling below the safe threshold. Nonetheless, it is quite common for energy intake to decrease, which can lead to malnutrition [33].

In comparison to a typical dietary protein intake of 0.8 g per kilogram per day, reducing dietary protein intake has been suggested as a possibility to decrease glomerular hyperfiltration and potentially decelerate the progression of CKD [34].

For diabetic patients without renal impairment, dietary protein requirements are assumed to be similar to those of the general population [35]. However, individuals with type 2 diabetes may often consume protein-rich foods more frequently for purposes such as promoting weight loss (e.g., Atkins and protein powder diets) or preventing hypoglycemia [36].

Even in more recent studies, it has been suggested that restricting dietary protein intake may have uncertain effects on changes in kidney function over time and may not significantly influence the risk of death or kidney failure [37].

Comparative analysis indicates that a restricted protein diet effectively slows the progression of CKD in non-diabetic individuals and those with type 1 diabetes with CKD. It appears that there is no significant benefit in terms of slowing CKD progression when protein restriction is implemented in CKD patients with type 2 diabetes [38].

For individuals with nephrotic syndrome, the guidelines suggest moderate protein intake to manage proteinuria and prevent further kidney damage. The current recommendation is approximately 0.8 to 1.0 g of protein per kilogram of body weight per day. This balance aims to reduce protein loss in urine while providing sufficient protein to prevent malnutrition, which is a common concern in nephrotic syndrome patients. The latest guidelines distinguish between predialysis patients with and without diabetes, offering specific protein intake ranges for each group. For clinically stable patients with CKD stages 3–5 without diabetes, the guidelines suggest a protein intake range of 0.55–0.60 g per kilogram per day, or alternatively, a very low-protein diet ranging from 0.28–0.43 g per kilogram per day supplemented with keto-acid analogs [39]. Nevertheless, it is important to emphasize that the beneficial effect of low-protein diets in DKD patients is still unclear and extremely restrictive protein intake may increase the risk of mortality. Many factors should be considered when a low or very low protein diet is recommended, such as associated comorbidities, the age of the patient, CKD stage. In 2013, the American Diabetes Association suggested for DKD patients daily protein intake similar to that of the healthy population (1–1.5 g/body weight) [40].

### 4.1. Plant Versus Animal Protein

The source of dietary protein may affect the progression of DKD. Animal protein is strongly linked to insulin resistance, and it increases the risk of hyperfiltration and albuminuria. In contrast, plant-sourced protein has a significantly beneficial effect on both DKD and cardiovascular disease (CVD) [41].

The mechanisms through which plant-based diets may decrease the progression of DKD could be attributed to several factors. These may include the form of phosphorus present in plant-based foods, a reduction in blood pressure through decreased sodium intake, an increase in fiber leading to better glycemic control, or the presence of bioactive compounds found in soy protein-based diets, such as isoflavones. Plant-based sources of protein can be either soy-based or non-soy-based. Soy-based diets have been shown to decrease urine albumin excretion, possibly due to the actions of isoflavones [42]. Plant-based sources of protein typically contain phosphorus in the less bioavailable form of phytate, whereas processed foods and animal sources of protein contain phosphorus in the organic form [43].

New findings suggest that the prevention of DKD progression can be achieved by recommending high intake of anthocyanins (ANT), which can determine a reduction in fasting blood glucose levels and the improvement of renal morphology and function. This intervention may be linked to adequate control of amino acid metabolism by enhancing the metabolism of tyrosine, tryptophan, taurine, and hypotaurine [44]. Ensuring sufficient intake of antioxidants through diet, as indicated by higher CDAI (Composite Dietary Antioxidant Index) levels, could potentially reduce the risk of DKD and mortality among individuals with diabetes. These results suggest a promising strategy for managing diabetes and underscore the potential of incorporating food-based antioxidants as a preventive measure (Table 1) [45,46].

### 4.2. The Effect of Ketoanalogues

The effects of ketoanalogues (KA) on CKD have not been fully confirmed. Ketoanalogues of amino acids represent nitrogen-free analogs of essential amino acids, and the “ketodiet” refers to low-protein diets (0.6 g/kg per day) or very-low-protein diets (0.3–0.4 g/kg per day) associated with KA [47]. DKD is characterized by systemic inflammation, insulin resistance, and hypercatabolism. Protein-restricted diets might lead to the risk of protein energy waste and essential amino acid (EAA) deficiency [48].

The National Kidney Foundation suggests a low-protein diet with 0.6–0.8 g/kg/day associated with adequate energy intake (25–35 kcal/kg/day) to preserve renal function and maintain nutritional status [49], while the American Diabetic Association considers that protein intake should not be lower than 0.8 g/kg/day due to the risk of malnutrition [50]. Because most major trials have not included DKD patients, mainly because of the fear of malnutrition, the potential clinical benefits and harms of KA supplementation with protein-restricted diets in non-dialysis DKD patients are not sufficiently characterized [51]. Effect sizes from ten randomized controlled trials (RCTs) and two non-RCTs, involving a total of 951 patients, were pooled and analyzed. The analysis revealed that a restricted protein diet supplemented with ketoanalogues (RPKA) had a major effect on the progression of CKD, particularly in patients with an eGFR greater than 18 mL/min/1.73 m^2^. Furthermore, RPKA did not cause malnutrition compared to the placebo. The results also suggested that ketoanalogues significantly reduce serum phosphorus levels in patients with poorer renal function, but cholesterol levels were not significantly influenced by KA [52].

This approach educates patients on proper nutrition from the early stages of the disease, preventing abrupt changes in eating habits. This gradual adjustment promotes better acclimatization and, consequently, improved adherence to dietary treatment, in accordance with patients’ eGFR value [24]:

- >60 mL/min, a normal protein intake is recommended (1–1.2 g proteins/kg/day);
- 45–59 mL/min, 0.8 g proteins/kg/day;
- 30–40 mL/min, 0.6–0.7 g proteins/kg/day;
- 15–29 mL/min, 0.6–0.7 g proteins/kg/day (phosphate awareness) or <0.6 g proteins/kg/day + ketoanalogues;
- <15 mL/min, 0.6 g proteins/kg/day (phosphate awareness) or 0.3–0.4 g proteins/kg/day + ketoanalogues.

## 5. Carbohydrate Intake

Obesity is linked to a heightened risk of CKD, emphasizing the importance of weight management in obese individuals with CKD. Guidance suggests that modest weight reduction, typically in the range of 5–10% of body weight, is advisable for obese CKD patients to help prevent the progression of kidney disease [41].

In patients with diabetic nephropathy, a diet low in both carbohydrates and proteins should be considered. It is difficult to choose one of the two nutritional therapies because there are studies showing that a low-protein diet could have several benefits, such as better control of blood pressure and glucose and cholesterol levels and may increase the patient’s life span [32].

The impact of a carbohydrate diet on outcomes in patients with DKD remains a matter of debate, considering that high intake can be linked to DKD onset [37]. Carbohydrates provide 45–60% of energy intake; therefore, in DKD patients, dietary sources should be carefully selected. Low glycemic index carbohydrates (e.g., fresh fruits and vegetables, fibers, etc.) should be mainly recommended, and fast-absorbing carbohydrates should be limited in order to provide less than 10% of the energy intake [53,54].

## 6. Effects of Omega-3 Fatty Acid Supplementation

In DKD, dyslipidemia treatment is essential in order to decrease the risk of cardiovascular events and premature mortality [55,56]. It is known that the risk of CVD in diabetic patients could be decreased by decreasing the intake of trans-fat and saturated fatty acids (SFA) [57].

In contrast, there are several studies that noticed the beneficial effects of omega-3 fatty acids (O3FAs), such as α-linoleic acid (ALA), eicosapentaenoic acid (EPA), and docosahexaenoic acid (DHA). These essential fatty acids are polyunsaturated fatty acids (PUFAs) and their main sources are fish, fresh seafood, seaweed, seed oil, vegetable oil, and nuts. It has been proven that low intake of O3FAs can favor normal growth and the adequate regulation of serum triglyceride levels [58,59,60,61,62]. Additionally, other studies have reported the antihypertensive effects of O3FAs. Considering that one of the major risk factors for CKD development is hypertension, it is expected that these fatty acids will represent an important dietary intervention in delaying CKD progression. Omega-3 and 6 polyunsaturated fatty acids and monounsaturated fatty acids (MUFAs) have a great impact on DKD outcomes through the attenuation of endothelial dysfunction and inflammation [63]. It is recommended that saturated fats be limited to <7% of total daily calories as, in excess, they can induce lipotoxicity [57,64,65]. There are studies that aimed to highlight the effect of long-chain omega-3 PUFAs on albuminuria in patients with DKD, but they only suggested the possibility of albuminuria decreasing. At the same time, there was insufficient evidence to exclude the effect of PUFAs on glomerular dysfunction [66,67].

## 7. Restriction of Dietary Sodium

In 2021, Kidney Disease Improving Global Outcomes (KDIGO) guidelines for glomerulonephritis recommended limiting dietary sodium intake to less than 2 g per day. This is essential for managing blood pressure, reducing edema, and enhancing urinary protein excretion, all without reliance on medication [68]. However, some studies have reported that excessively low sodium intake adversely affects glucose metabolism and decreases insulin sensitivity. In addition, the activation of RAAS and the sympathetic nervous system following low dietary sodium may further reduce insulin sensitivity [69]. There are different studies that showed an increase in insulin resistance after only 1 week of a low-sodium diet. This inverse correlation is determined by increased synthesis of aldosterone and norepinephrine in response to low blood sodium; these hormones may lead to increased insulin resistance. In addition, high levels of angiotensin II noticed in low-sodium diets may contribute to a further increase in insulin resistance [70,71]. A review of 23 human clinical trials, focused on the effects of low-sodium diets on insulin, concluded that insulin resistance may be caused by the activation of the sympathetic nervous system and the renin–angiotensin–aldosterone system, the increase in intestinal glucose absorption secondary to a lower intracellular sodium level, and the presence of hypovolemia that leads to the decreased delivery of insulin or glucose into the skeletal muscles [71].

It remains uncertain whether these effects lead to a substantial overall risk reduction in patients with CKD, including the progression of renal impairment or cardiovascular events, and all-cause mortality [66]. Based on the evidence, it appears that a restrictive low-sodium diet may be linked to increased insulin resistance, activation of the renin–angiotensin–aldosterone system, and impaired renal function [71]. Additionally, from our experience, especially in older patients with advanced stages of CKD, restrictive sodium intake can lead to very low levels of blood sodium that can cause important neurological complications and increase the risk of mortality, even in the presence of adequate treatment management.

## 8. Potassium’s Role in DKD Patients

During the evolution of DKD, a decrease in potassium excretion is noticed, leading to its accumulation in body tissues, with a direct impact on muscle normal activity, as this electrolyte is essential in the contraction and relaxation of muscles. Foods with high potassium content, such as bananas, kiwi, melons, tomato juice, potatoes, mushrooms, beans, avocados, and spinach, should be avoided in patients with hyperkalemia. There are different methods to prevent potassium accumulation [72,73]:

- Using leaves, not stems, and removing the shell;
- Before cooking vegetables and coarse grains, soaking them in water for 2 to 3 h and then blanching them in boiling water;
- Using fruits and vegetables with low potassium content;
- Limiting the intake of processed foods, which in general present a high phosphorus content;
- When appropriate, recommending phosphorus-binding drugs.

Besides their significant potassium content, vegetables and fruits can increase fluid intake. Therefore, in patients with decreased urinary volume, this type of food should be seldom used and those with high water content should be avoided. Another recommendation is to drain them well after these products are washed.

## 9. Comparison between DASH and the Mediterranean Diet

The management of patients with diabetic nephropathy can be improved by implementing diets such as Dietary Approaches to Stop Hypertension (DASH) and the Mediterranean diet (MED) which both emphasize higher intake of vegetables, whole grains (e.g., complex and unrefined carbohydrates), and plant-based protein (e.g., nuts, seeds, and beans). There are studies on the DASH diet that have suggested a favorable impact on blood pressure and the incidence of diabetes, although it is not known whether this is due to its protein content or other components [65,74].

Given that the DASH diet prescribes a higher level of dietary protein intake, the application of this diet to DKD non-dialyzed patients should be modified. An RCT of the DASH diet (containing 18% energy from protein) versus a control diet administered over eight weeks did not show improvement in albuminuria, while reductions were seen in those who received a fruit and vegetable diet [75]. There are other aspects, such as the high phosphorus and potassium content of the recommended foods, that limit the use of the DASH diet in DKD patients.

**Figure 1:**
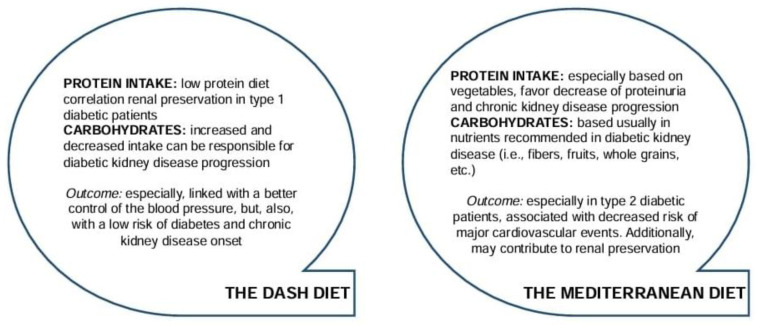
Known aspects of the DASH and Mediterranean diets’ impact on diabetic kidney disease. Modified according to reference [80].

The MED recommends high intake of olive oil and plant foods, such as unrefined cereals, significant amounts of vegetables, fresh fruits, etc. Despite the increased fat consumption, many studies have shown that this diet leads to a lower incidence of major cardiovascular events and type 2 diabetes [76]. There are studies that have shown the protective role of the MED in lowering HbA1c, controlling blood sugar, reducing insulin resistance, and lowering fasting blood sugar levels and mortality [77]. The Mediterranean diet is also associated with a lower risk of metabolic syndrome in post-transplant patients [78]. A 15-year observational study demonstrated that adherence to the Mediterranean diet was associated with a lower risk of rapid decline in eGFR [79]. To validate these findings and improve patient care, further studies regarding the DASH and Mediterranean diet in DKD patients are required (Figure 1) [80].

It should be emphasized that these diets based on plant proteins can also increase oxalate and phytate intake, which favors the risk of nephrolithiasis and nephrocalcinosis. Evidence suggests that only a 5 mg increase in oxalate excretion can substantially increase the risk of kidney stone development. Therefore, patients should be aware of foods with high content of oxalates and phytates (e.g., spinach, rhubarb, tofu, almonds, amaranth, cocoa powder, cereal grains, oilseeds, etc.) and avoid them. A balanced calcium diet is also recommended in order to reduce urinary oxalate and phytate excretion [81,82].

## 10. Inflammation in DKD Patients

DKD is often associated with increased levels of advanced glycation products (AGE) due to decreased renal excretion and increased oxidative stress secondary to the presence of hyperglycemia. It has been suggested that adequate fiber intake can reduce the levels of these products, leading to a decreased risk of cardiovascular events and mortality in this group of patients [83]. Once the AGE pathway is activated, along with protein kinase C and polyol pathways, increased expression of inflammatory cytokines (e.g., Toll-like receptors, interleukin-1β, interleukin-18, etc.) is noted, leading to proteinuria, insulin resistance, and renal fibrosis. Reactive oxygen species (ROS) accumulation represents another pathophysiological mechanism involved in the onset and progression of renal impairment. ROS upregulates TGF-β expression, which is involved in renal fibrosis onset and progression. Autophagy represents an important energy source and regulator mechanism to maintain adequate intracellular homeostasis. In DKD, mTOR complex 1 (a regulator of autophagy) is highly expressed and inhibits this process, which favors the presence of proteinuria and the development and progression of CKD. It seems that this inhibition can be reversed by the overexpression of sirtuin 1 (SIRT1), and oral supplementation with resveratrol, a SIRT1 activator, may be beneficial [84].

Furthermore, CKD patients are often associated with gut dysbiosis due to increased production of uremic toxins. The integrity of the intestinal barrier is compromised by the increase in pH value in the intestinal lumen, caused by the presence of elevated serum urea levels, which is converted to ammonia and then to ammonium hydroxide. Furthermore, the presence of gut dysbiosis is associated with an increased concentration of bacteria responsible for toxin precursors that increase oxidative stress. In addition, these toxins (e.g., p-cresyl sulfate and indoxyl sulfate) reduce the number of epithelial layers, favoring the transit of endotoxins, and, consequently, the onset of renal fibrosis. Evidence suggests that increased fiber intake can provide indigestible starch that stimulates motility and favors the growth of helpful bacteria. Therefore, fiber diets can improve renal function, decrease the time for amino acid fermentation into uremic solutes, and also decrease inflammation and oxidation, leading to better metabolic control. There are studies that have reported that a diet based on fruits, whole grains, and vegetables was not only able to decrease the risk of CKD but also the risk of all-cause mortality. The integrity of the intestinal barrier can also be maintained by increased production of short-chain fatty acids (SCFAs). A fiber-rich diet can increase this production, which favors amino acids to be incorporated into the bacterial proteins of the colon; this process leads to the excretion of the amino acids instead of them being fermented into uremic toxins [83,85]. Several studies note the protective effects of SCFAs in DKD patients [84]:

- Anti-inflammatory effects by decreasing proinflammatory factors (e.g., interleukin-1, inteleukin-6, etc.) and inhibiting the nuclear factor kappa beta pathway;
- The reduction in ROS generation, leading to a decreased oxidative stress state;
- The improvement of energy metabolism through the increase in insulin sensitivity and the loss of body weight;
- The maintenance of podocyte structural integrity which favors the decrease in proteinuria and, consequently, the improvement of renal function.

## 11. Conclusions

Chronic kidney disease presents a growing concern regarding patients’ quality of life and often leads to significant mortality, particularly in its advanced stages. The therapeutic management of DKD is a multifaceted issue, encompassing both nutritional therapy and pharmacotherapy. The diet plan for DKD patients involves meticulous adjustment of macro and micronutrients, crucial for disease prognosis and progression. Nutritional status emerges as a modifiable factor influencing the evolution of DKD, emphasizing the importance of preventing malnutrition while adhering to dietary restrictions essential for disease management.

Despite significant progress in understanding and managing DKD, uncertainties persist, particularly regarding optimal dietary strategies and their impact on long-term outcomes. Therefore, it is necessary for a personalized diet to be recommended for each patient, as in the final stages of CKD, foods with high content of potassium, phosphate, and water should be avoided. Future studies are needed to elucidate the intricate interactions between diet, disease progression, and patient outcomes in DKD, paving the way for more effective therapeutic interventions and the improvement of patient care.

## Figures and Tables

**Table 1:** Comparison between animal and plant protein in diabetic kidney disease.

Source of Protein	Benefits	Risks
Plants (e.g., almonds, black beans, oats, tofu, chickpeas, red lentils, spinach, broccoli, edamame, etc.)	increase in fiber leading to better glycemic control contain vitamins and minerals no cholesterol contain phosphorus in the less bioavailable form of phytate lower risk of heart disease, diabetes, and DKD	lower nutritional values due to unbalanced amino acid composition incomplete protein profiles lower digestibility digestive issues such as bloating, gas, and discomfort in some individuals
Animals (e.g., chicken, turkey, salmon, tuna, egg, yogurt, duck, prawns, ribeye, etc.)	contribute to increased lean muscle mass prevent malnutrition contain all essential amino acids higher in vitamin B12 and iron help in the absorption of other nutrients, e.g., casein micelles help in the absorption of proteins, calcium, and phosphate in high concentrations	higher saturated fat high cholesterol low dietary fiber higher calories increase the risk of hyperfiltration and albuminuria contain phosphorus in organic form insulin resistance

## Data Availability

Data sharing is not applicable to this article.

## References

[1] Hill N.R., Fatoba S.T., Oke J.L., Hirst J.A., O’Callaghan C.A., Lasserson D.S., Hobbs F.D. (2016). Global Prevalence of Chronic Kidney Disease—A Systematic Review and Meta-Analysis. PLoS ONE.

[2] Tsai J.L., Chen C.H., Wu M.J., Tsai S.F. (2022). New Approaches to Diabetic Nephropathy from Bed to Bench. Biomedicines.

[3] Zhang L., Long J., Jiang W., Shi Y., He X., Zhou Z., Li Y., Yeung R.O., Wang J., Matsushita K. (2016). Trends in Chronic Kidney Disease in China. N. Engl. J. Med..

[4] Umanath K., Lewis J.B. (2018). Update on Diabetic Nephropathy: Core Curriculum 2018. Am. J. Kidney Dis..

[5] Sulaiman M.K. (2019). Diabetic nephropathy: Recent advances in pathophysiology and challenges in dietary management. Diabetol. Metab. Syndr..

[6] International Diabetes Federation (2015). International Diabetes Federation IDF Diabetes Atlas.

[7] Jansson Sigfrids F., Groop P.H. (2023). Progression and regression of kidney disease in type 1 diabetes. Front. Nephrol..

[8] Szczech L.A., Stewart R.C., Su H.L., DeLoskey R.J., Astor B.C., Fox C.H., McCullough P.A., Vassalotti J.A. (2014). Primary care detection of chronic kidney disease in adults with type-2 diabetes: The ADD-CKD Study (awareness, detection and drug therapy in type 2 diabetes and chronic kidney disease). PLoS ONE.

[9] Zhang J., Liu J., Qin X. (2018). Advances in early biomarkers of diabetic nephropathy. Rev. Assoc. Med. Bras. (1992).

[10] Adler A.I., Stevens R.J., Manley S.E., Bilous R.W., Cull C.A., Holman R.R., UKPDS Group (2003). Development and progression of nephropathy in type 2 diabetes: The United Kingdom Prospective Diabetes Study (UKPDS 64). Kidney Int..

[11] Cooper M.E. (2012). Diabetes: Treating diabetic nephropathy-still an unresolved issue. Nat. Rev. Endocrinol..

[12] Natesan V., Kim S.J. (2021). Diabetic Nephropathy—A Review of Risk Factors, Progression, Mechanism, and Dietary Management. Biomol. Ther..

[13] Papadopoulou-Marketou N., Paschou S.A., Marketos N., Adamidi S., Adamidis S., Kanaka-Gantenbein C. (2018). Diabetic nephropathy in type 1 diabetes. Minerva Med..

[14] Papadopoulou-Marketou N., Kanaka-Gantenbein C., Marketos N., Chrousos G.P., Papassotiriou I. (2017). Biomarkers of diabetic nephropathy: A 2017 update. Crit. Rev. Clin. Lab. Sci..

[15] Meloni C., Tatangelo P., Cipriani S., Rossi V., Suraci C., Tozzo C., Rossini B., Cecilia A., Di Franco D., Straccialano E. (2004). Adequate protein dietary restriction in diabetic and nondiabetic patients with chronic renal failure. J. Ren. Nutr..

[16] Oltean S., Coward R., Collino M., Baelde H. (2017). Diabetic Nephropathy: Novel Molecular Mechanisms and Therapeutic Avenues. BioMed Res. Int..

[17] Montero R.M., Covic A., Gnudi L., Goldsmith D. (2016). Diabetic nephropathy: What does the future hold?. Int. Urol. Nephrol..

[18] Lytvyn Y., Bjornstad P., Pun N., Cherney D.Z. (2016). New and old agents in the management of diabetic nephropathy. Curr. Opin. Nephrol. Hypertens..

[19] Siddiqui K., George T.P., Joy S.S., Alfadda A.A. (2022). Risk factors of chronic kidney disease among type 2 diabetic patients with longer duration of diabetes. Front. Endocrinol..

[20] Gross J.L., de Azevedo M.J., Silveiro S.P., Canani L.H., Caramori M.L., Zelmanovitz T. (2005). Diabetic nephropathy: Diagnosis, prevention, and treatment. Diabetes Care.

[21] Kim J.Y. (2013). Diet therapy in patients of diabetic nephropathy. J. Korean Diabetes.

[22] Bach K.E., Kelly J.T., Palmer S.C., Khalesi S., Strippoli G.F.M., Campbell K.L. (2019). Healthy Dietary Patterns and Incidence of CKD: A Meta-Analysis of Cohort Studies. Clin. J. Am. Soc. Nephrol..

[23] Kim H.Y. (2014). Nutritional intervention for a patient with diabetic nephropathy. Clin. Nutr. Res..

[24] Bellizzi V. (2013). Low-protein diet or nutritional therapy in chronic kidney disease?. Blood Purif..

[25] Cai L., Huang Y., Li X., Cao D., Liu F. (2024). Effects of dietary intervention on diabetic nephropathy: An umbrella review of systematic reviews and meta-analyses of randomized controlled trials. Front. Endocrinol..

[26] Jamale T., Bose S. (2024). To restrict or not to restrict—Understanding the conundrum of dietary protein restriction in chronic kidney disease. J. Postgrad. Med..

[27] Obeid W., Hiremath S., Topf J.M. (2022). Protein Restriction for CKD: Time to Move On. Kidney360.

[28] Cecchi S., Di Stante S., Belcastro S., Bertuzzi V., Cardillo A., Diotallevi L., Grabocka X., Kulurianu H., Martello M., Nastasi V. (2023). Supplemented Very Low Protein Diet (sVLPD) in Patients with Advanced Chronic Renal Failure: Clinical and Economic Benefits. Nutrients.

[29] Capitanio C., Terraneo V., Milia V., Pozzi C. (2019). The low-protein diet for chronic kidney disease: 8 years of clinical experience in a nephrology ward. Clin. Kidney J..

[30] Langsetmo L., Harrison S., Jonnalagadda S., Pereira S.L., Shikany J.M., Farsijani S., Lane N.E., Cauley J.A., Stone K., Cawthon P.M. (2020). Low Protein Intake Irrespective of Source is Associated with Higher Mortality Among Older Community-dwelling Men. J. Nutr. Health Aging.

[31] Mitch W.E., Remuzzi G. (2004). Diets for patients with chronic kidney disease, still worth prescribing. J. Am. Soc. Nephrol..

[32] Hanna R.M., Ghobry L., Wassef O., Rhee C.M., Kalantar-Zadeh K. (2020). A Practical Approach to Nutrition, Protein-Energy Wasting, Sarcopenia, and Cachexia in Patients with Chronic Kidney Disease. Blood Purif..

[33] Bellizzi V., Garofalo C., Ferrara C., Calella P. (2022). Ketoanalogue Supplementation in Patients with Non-Dialysis Diabetic Kidney Disease: A Systematic Review and Meta-Analysis. Nutrients.

[34] Nguyen D.V., Kalantar-Zadeh K., Rhee C.M. (2021). Dietary protein intake, kidney function, and survival in a nationally representative cohort. Am. J. Clin. Nutr..

[35] Gregg E.W., Li Y., Wang J., Burrows N.R., Ali M.K., Rolka D., Williams D.E., Geiss L. (2014). Changes in diabetes-related complications in the United States, 1990–2010. N. Engl. J. Med..

[36] Campbell A.P., Rains T.M. (2015). Dietary protein is important in the practical management of prediabetes and type 2 diabetes. J. Nutr..

[37] Jiang S., Fang J., Li W. (2023). Protein restriction for diabetic kidney disease. Cochrane Database Syst. Rev..

[38] Rughooputh M.S., Zeng R., Yao Y. (2015). Protein Diet Restriction Slows Chronic Kidney Disease Progression in Non-Diabetic and in Type 1 Diabetic Patients, but Not in Type 2 Diabetic Patients: A Meta-Analysis of Randomized Controlled Trials Using Glomerular Filtration Rate as a Surrogate. PLoS ONE.

[39] Kistler B.M., Moore L.W., Benner D., Biruete A., Boaz M., Brunori G., Chen J., Drechsler C., Guebre-Egziabher F., Hensley M.K. (2021). The International Society of Renal Nutrition and Metabolism Commentary on the National Kidney Foundation and Academy of Nutrition and Dietetics KDOQI Clinical Practice Guideline for Nutrition in Chronic Kidney Disease. J. Ren. Nutr..

[40] Yamada S. (2021). Do No Harm: Critical Appraisal of Protein Restriction for Diabetic Kidney Disease. Diabetology.

[41] Adeva-Andany M.M., Fernández-Fernández C., Carneiro-Freire N., Vila-Altesor M., Ameneiros-Rodríguez E. (2022). The differential effect of animal versus vegetable dietary protein on the clinical manifestations of diabetic kidney disease in humans. Clin. Nutr. ESPEN.

[42] Moorthi R.N., Vorland C.J., Hill Gallant K.M. (2017). Diet and Diabetic Kidney Disease: Plant Versus Animal Protein. Curr. Diab. Rep..

[43] Moe S.M., Zidehsarai M.P., Chambers M.A., Jackman L.A., Radcliffe J.S., Trevino L.L., Donahue S.E., Asplin J.R. (2011). Vegetarian compared with meat dietary protein source and phosphorus homeostasis in chronic kidney disease. Clin. J. Am. Soc. Nephrol..

[44] Li Y.X., Lu Y.P., Tang D., Hu B., Zhang Z.Y., Wu H.W., Fan L.J., Cai K.W., Tangm C., Zhang Y.Q. (2022). Anthocyanin improves kidney function in diabetic kidney disease by regulating amino acid metabolism. J. Transl. Med..

[45] Zhang J., Chen Y., Zou L., Jin L., Yang B., Shu Y., Gong R. (2023). Dose-response relationship between dietary antioxidant intake and diabetic kidney disease in the US adults with diabetes. Acta Diabetol..

[46] Hoffman J.R., Falvo M.J. (2004). Protein—Which is Best?. J. Sports Sci. Med..

[47] Khan I.A., Nasiruddin M., Haque S.F., Khan R.A. (2016). Comparative evaluation of efficacy and safety profile of rhubarb and α-keto analogs of essential amino acids supplementation in patients with diabetic nephropathy. Saudi J. Kidney Dis. Transpl..

[48] Chang J.H., Kim D.K., Park J.T., Kang E.W., Yoo T.H., Kim B.S., Choi K.H., Lee H.Y., Han D.S., Shin S.K. (2009). Influence of ketoanalogs supplementation on the progression in chronic kidney disease patients who had training on low-protein diet. Nephrology (Carlton).

[49] Ikizler T.A., Burrowes J.D., Byham-Gray L.D., Campbell K.L., Carrero J.J., Chan W., Fouque D., Friedman A.N., Ghaddar S., Goldstein-Fuchs D.J. (2020). KDOQI Clinical Practice Guideline for Nutrition in CKD: 2020 Update. Am. J. Kidney Dis..

[50] American Diabetes Association (2019). 2. Classification and Diagnosis of Diabetes: Standards of Medical Care in Diabetes-2019. Diabetes Care.

[51] Bellizzi V., Signoriello S., Minutolo R., Di Iorio B., Nazzaro P., Garofalo C., Calella P., Chiodini P., De Nicola L., ERIKA Study Group Investigators of the Italian Society of Nephrology-Conservative Therapy of CKD Work Group (2022). No additional benefit of prescribing a very low-protein diet in patients with advanced chronic kidney disease under regular nephrology care: A pragmatic, randomized, controlled trial. Am. J. Clin. Nutr..

[52] Li A., Lee H.Y., Lin Y.C. (2019). The Effect of Ketoanalogues on Chronic Kidney Disease Deterioration: A Meta-Analysis. Nutrients.

[53] KDOQI (2007). KDOQI Clinical Practice Guidelines and Clinical Practice Recommendations for Diabetes and Chronic Kidney Disease. Am. J. Kidney Dis..

[54] Tuttle K.R., Bakris G.L., Bilous R.W., Chiang J.L., de Boer I.H., Goldstein-Fuchs J., Hirsch I.B., Kalantar-Zadeh K., Narva A.S., Navaneethan S.D. (2014). Diabetic kidney disease: A report from an ADA Consensus Conference. Diabetes Care.

[55] Michas G., Micha R., Zampelas A. (2014). Dietary fats and cardiovascular disease: Putting together the pieces of a complicated puzzle. Atherosclerosis.

[56] Anderson T.J., Grégoire J., Hegele R.A., Couture P., Mancini G.B., McPherson R., Francis G.A., Poirier P., Lau D.C., Grover S. (2013). 2012 update of the Canadian Cardiovascular Society guidelines for the diagnosis and treatment of dyslipidemia for the prevention of cardiovascular disease in the adult. Can. J. Cardiol..

[57] Ley S.H., Hamdy O., Mohan V., Hu F.B. (2014). Prevention and management of type 2 diabetes: Dietary components and nutritional strategies. Lancet.

[58] Nomali M., Heidari M.E., Ayati A., Tayebi A., Shevchuk O., Mohammadrezaei R., Navid H., Khayyatzadeh S.S., Palii S., Valizade Shiran F. (2024). Omega-3 supplementation and outcomes of heart failure: A systematic review of clinical trials. Medicine.

[59] Froyen E., Burns-Whitmore B. (2020). The Effects of Linoleic Acid Consumption on Lipid Risk Markers for Cardiovascular Disease in Healthy Individuals: A Review of Human Intervention Trials. Nutrients.

[60] Davidson M.H., Stein E.A., Bays H.E., Maki K.C., Doyle R.T., Shalwitz R.A., Ballantyne C.M., Ginsberg H.N., COMBination of prescription Omega-3 with Simvastatin (COMBOS) Investigators (2007). Efficacy and tolerability of adding prescription omega-3 fatty acids 4 g/d to simvastatin 40 mg/d in hypertriglyceridemic patients: An 8-week, randomized, double-blind, placebo-controlled study. Clin. Ther..

[61] Bucher H.C., Hengstler P., Schindler C., Meier G. (2002). N-3 polyunsaturated fatty acids in coronary heart disease: A meta-analysis of randomized controlled trials. Am. J. Med..

[62] Zhang X., Ritonja J.A., Zhou N., Chen B.E., Li X. (2022). Omega-3 Polyunsaturated Fatty Acids Intake and Blood Pressure: A Dose-Response Meta-Analysis of Randomized Controlled Trials. J. Am. Heart Assoc..

[63] Shapiro H., Theilla M., Attal-Singer J., Singer P. (2011). Effects of polyunsaturated fatty acid consumption in diabetic nephropathy. Nat. Rev. Nephrol..

[64] Salas-Salvadó J., Martinez-González M.Á., Bulló M., Ros E. (2011). The role of diet in the prevention of type 2 diabetes. Nutr. Metab. Cardiovasc. Dis..

[65] Tyson C.C., Nwankwo C., Lin P.H., Svetkey L.P. (2012). The Dietary Approaches to Stop Hypertension (DASH) eating pattern in special populations. Curr. Hypertens. Rep..

[66] Donadio J.V., Grande J.P. (2004). The role of fish oil/omega-3 fatty acids in the treatment of IgA nephropathy. Semin. Nephrol..

[67] Friedman A., Moe S. (2006). Review of the effects of omega-3 supplementation in dialysis patients. Clin. J. Am. Soc. Nephrol..

[68] Kidney Disease: Improving Global Outcomes (KDIGO) Diabetes Work Group (2022). KDIGO 2022 Clinical Practice Guideline for Diabetes Management in Chronic Kidney Disease. Kidney Int..

[69] Kong Y.W., Baqar S., Jerums G., Ekinci E.I. (2016). Sodium and Its Role in Cardiovascular Disease—The Debate Continues. Front. Endocrinol..

[70] Garg R., Williams G.H., Hurwitz S., Brown N.J., Hopkins P.N., Adler G.K. (2011). Low-salt diet increases insulin resistance in healthy subjects. Metabolism.

[71] DiNicolantonio J.J., O’Keefe J.H. (2023). Sodium restriction and insulin resistance: A review of 23 clinical trials. J. Insul. Resist..

[72] Choi J.H., Lee K.A., Moon J.H., Chon S., Kim D.J., Kim H.J., Kim N.H., Seo J.A., Kim M.K., Lim J.H. (2023). 2023 Clinical Practice Guidelines for Diabetes Mellitus of the Korean Diabetes Association. Diabetes Metab. J..

[73] Goldstein-Fuchs J., Kalantar-Zadeh K. (2015). Nutrition Intervention for Advanced Stages of Diabetic Kidney Disease. Diabetes Spectr..

[74] Chiavaroli L., Viguiliouk E., Nishi S.K., Blanco Mejia S., Rahelić D., Kahleová H., Salas-Salvadó J., Kendall C.W., Sievenpiper J.L. (2019). DASH Dietary Pattern and Cardiometabolic Outcomes: An Umbrella Review of Systematic Reviews and Meta-Analyses. Nutrients.

[75] Jacobs D.R., Gross M.D., Steffen L., Steffes M.W., Yu X., Svetkey L.P., Appel L.J., Vollmer W.M., Bray G.A., Moore T. (2009). The effects of dietary patterns on urinary albumin excretion: Results of the Dietary Approaches to Stop Hypertension (DASH) Trial. Am. J. Kidney Dis..

[76] Salas-Salvadó J., Bulló M., Babio N., Martínez-González M.Á., Ibarrola-Jurado N., Basora J., Estruch R., Covas M.I., Corella D., Arós F. (2011). Reduction in the incidence of type 2 diabetes with the Mediterranean diet: Results of the PREDIMED-Reus nutrition intervention randomized trial. Diabetes Care.

[77] American Diabetes Association (2018). 4. Lifestyle Management: Standards of Medical Care in Diabetes-2018. Diabetes Care.

[78] Nafar M., Noori N., Jalali-Farahani S., Hosseinpanah F., Poorrezagholi F., Ahmadpoor P., Samadian F., Firouzan A., Einollahi B. (2009). Mediterranean diets are associated with a lower incidence of metabolic syndrome one year following renal transplantation. Kidney Int..

[79] Khatri M., Moon Y.P., Scarmeas N., Gu Y., Gardener H., Cheung K., Wright C.B., Sacco R.L., Nickolas T.L., Elkind M.S. (2014). The association between a Mediterranean-style diet and kidney function in the Northern Manhattan Study cohort. Clin. J. Am. Soc. Nephrol..

[80] Ko G.J., Kalantar-Zadeh K., Goldstein-Fuchs J., Rhee C.M. (2017). Dietary Approaches in the Management of Diabetic Patients with Kidney Disease. Nutrients.

[81] Bargagli M., Tio M.C., Waikar S.S., Ferraro P.M. (2020). Dietary Oxalate Intake and Kidney Outcomes. Nutrients.

[82] Kim O.H., Booth C.J., Choi H.S., Lee J., Kang J., Hur J., Jung W.J., Jung Y.S., Choi H.J., Kim H. (2020). High-phytate/low-calcium diet is a risk factor for crystal nephropathies, renal phosphate wasting, and bone loss. eLife.

[83] Cigarrán Guldris S., Latorre Catalá J.A., Sanjurjo Amado A., Menéndez Granados N., Piñeiro Varela E. (2022). Fibre Intake in Chronic Kidney Disease: What Fibre Should We Recommend?. Nutrients.

[84] Tao P., Ji J., Wang Q., Cui M., Cao M., Xu Y. (2022). The role and mechanism of gut microbiota-derived short-chain fatty in the prevention and treatment of diabetic kidney disease. Front. Immunol..

[85] Lochmann H. (2022). The Benefits of Fiber in Chronic Kidney Disease. J. Ren. Nutr..

